# Internal consistency of the Strengths and Difficulties Questionnaire in Amazonian children

**DOI:** 10.11606/s1518-8787.2023057005562

**Published:** 2024-02-01

**Authors:** Isabel Giacomini, Maria Rosário O. Martins, Alicia Matijasevich, Marly A. Cardoso

**Affiliations:** I Universidade de São Paulo Faculdade de Saúde Pública São Paulo SP Brasil Universidade de São Paulo. Faculdade de Saúde Pública. Programa de Pós-Graduação em Nutrição em Saúde Pública. São Paulo, SP, Brasil; II Universidade NOVA de Lisboa Instituto de Higiene e Medicina Tropical Lisboa Portugal Universidade NOVA de Lisboa. Instituto de Higiene e Medicina Tropical, Saúde Global e Medicina Tropical. Lisboa, Portugal; III Universidade de São Paulo Faculdade de Medicina Departamento de Medicina Preventiva São Paulo SP Brasil Universidade de São Paulo. Faculdade de Medicina. Departamento de Medicina Preventiva. São Paulo, SP, Brasil; IV Universidade de São Paulo Faculdade de Saúde Pública Departamento de Nutrição São Paulo SP Brasil Universidade de São Paulo. Faculdade de Saúde Pública. Departamento de Nutrição. São Paulo, SP, Brasil

**Keywords:** Psychometrics, Problem Behavior, Child Behavior Disorders, Behavior Rating Scale

## Abstract

**OBJECTIVE:**

To describe the frequency of behavioral problems and the internal consistency of the parent version of the Strengths and Difficulties Questionnaire (SDQ-P) in Amazonian preschool children during the covid-19 pandemic.

**METHODS:**

Data from the Maternal and Child Health and Nutrition in Acre (MINA-Brazil) study, a population-based birth cohort in the Western Brazilian Amazon, were used. The SDQ-P was applied in 2021 at the five-year follow-up visit to parents or caregivers of 695 children (49.4% of which were girls). This instrument is a short behavioral screening questionnaire composed of 25 items reorganized into five subscales: emotional symptoms, conduct problems, hyperactivity/inattention, peer relationship problems, and prosocial behavior. Cases of behavioral problems were defined according to the original SDQ cut-offs based on United Kingdom norms. Moreover, cut off points were estimated based on the SDQ-P percentile results of our study sample. Internal consistency was assessed by calculating Cronbach's alpha coefficient and McDonald's omega for each scale.

**RESULTS:**

According to the cut-offs based on our studied population distribution, 10% of all children had high or very high total difficulty scores, whereas it was almost twice when the original SDQ cut-offs based on United Kingdom norms, were applied (18%). Differences were also observed in the other scales. Compared to girls, boys showed higher means of externalizing problem and lower means of prosocial behavior. The five-factor model showed a moderate internal consistency of the items for all scales (0.60 ≤ α ≤ 0.40), except for total difficulty scores, which it considered substantial (α > 0.61).

**CONCLUSIONS:**

Our results support the usefulness of SDQ in our study population and reinforce the need for strategies and policy development for mental health care in early life in the Amazon.

## INTRODUCTION

Estimates suggest that about 20% of all children in the world under the age of seven years experience some type of mental disorder^1^. Thus, addressing mental health and precocious psychosocial development of children concerns both their chance of achieving a fulfilling life and the formation of human capital. Early childhood represents a particularly critical and vulnerable period as children in their early years of life respond to traumas and stress or express their emotions and feelings in specific ways unlike those of older children^1,2^.

The frequency of mental health disorders during childhood varies between countries. Data from children aged between 36 and 59 months from 63 countries showed that the frequency of suspected social-emotional delay is heterogeneous, ranging from 20% in high-income countries to 18%, 25%, and 32% in upper middle-, lower middle-, and low-income countries, respectively^2^.

The Strengths and Difficulties Questionnaire (SDQ) aims to track behavioral problems in children aged 2–17 years^3^. It is available in more than 40 languages and in three versions: for teachers (SDQ-T), parents/guardians (SDQ-P), and self-report (SDQ-S). It includes 25 questions about children's behaviors or abilities that are organized into five scales: emotional symptoms, conduct problems, hyperactivity, peer problems, and prosocial behavior.

SDQ results can be explored in two ways: as raw scores or categories, with cut-off points proposed by the authors of the instrument. Bands were established according to a large population-based study in the United Kingdom (UK), in which 80% of the children were classified as "close to average"; 10%, as "slightly raised"; 5%, as "high"; and 5%, as "very high" regarding behavioral problems in each scale (except for the prosocial scale, which is scored in reverse). However, considering the socioeconomic and cultural differences across countries, studies outside Europe suggested that bandings based on each sample distribution should be considered for comparisons^4^.

More recently, as an alternative to the aforementioned five-scale model, a new three-factor solution has been proposed for low-risk or general population samples^5^. This model reorganizes SDQ items into three subscales: internalizing problems (the sum of emotional and peer symptoms), externalizing problems (the sum of conduct and hyperactivity symptoms), and prosocial behavior^5^.

The SDQ was validated in Brazil using a two-phase assessment in different local contexts to evaluate its retest reliability^6–8^. It is easy to implement and, when prior training is provided to users, a specialized professional is not required^8^. Furthermore, as the scientific community extensively uses the SDQ, results can be compared between national and international studies^9,10^.

Although the SDQ has been applied in different Brazilian sites, it has been scarcely used in the remote areas of the country and its internal consistency has not been assessed. In the Brazilian Amazon, children in the most socioeconomically vulnerable families were disproportionately affected by SARS-CoV-2 infections^11^. The great social inequalities and vulnerabilities require data on early childhood behavior in the Amazon. Therefore, this study describes the frequency of behavior disorders in preschool children during the COVID-19 restrictions. This study assessed this using the SDQ-P scales according to its original quadruple categorization and analyzed the internal consistency of the five- and three-solution models proposed by Goodman^3^ and Goodman et al.^5^, respectively, in a birth cohort in the Western Brazilian Amazon.

## METHODS

### Study Design, Site, and Population

The Maternal and Child Health and Nutrition in Acre, Brazil (MINA-Brazil Study), is a population-based birth cohort that was conducted in the municipality of Cruzeiro do Sul, Acre State, Western Brazilian Amazon. In 2021, Cruzeiro do Sul had an estimated population of 89,760 individuals (about 70% of whom lived in urban areas)^12^. The municipality represents the second largest municipal gross domestic product in Acre State and has a Human Development Index of 0.664^12^, which is below the Brazilian national average^13^.

The Institutional Review Board of the School of Public Health, Universidade de São Paulo, approved this study (# 872.613, 2014; # 2.358.129, 2017). Informed consent forms were obtained from all participating mothers or children's parents or guardians (if mothers were aged < 18 years).

### Baseline and Follow-Up Assessments

In 2015, mother-infant pairs were enrolled in the study either in public antenatal clinics or at birth in the Women and Children's Hospital of Juruá Valley, the only maternity hospital in Cruzeiro do Sul, as described elsewhere^14^. At baseline, 1,246 mother-infant pairs were eligible for follow-up visits. At birth, mothers' sociodemographic information was obtained, such as self-reported skin color (white, mixed, yellow, black, or amerindian); marital status (living with a partner or not); educational attainment (years of study); and beneficiary status of the *Bolsa Família* Program, a conditional cash transfer program directed to families living in poverty (yes or no)^15^. Obstetric and perinatal data (birth weight in grams, gestational age, and type of birth) were collected from maternity records. After the baseline assessment, follow-up visits were carried out at ages one, two, and five years (Figure).

**Figure 1:**
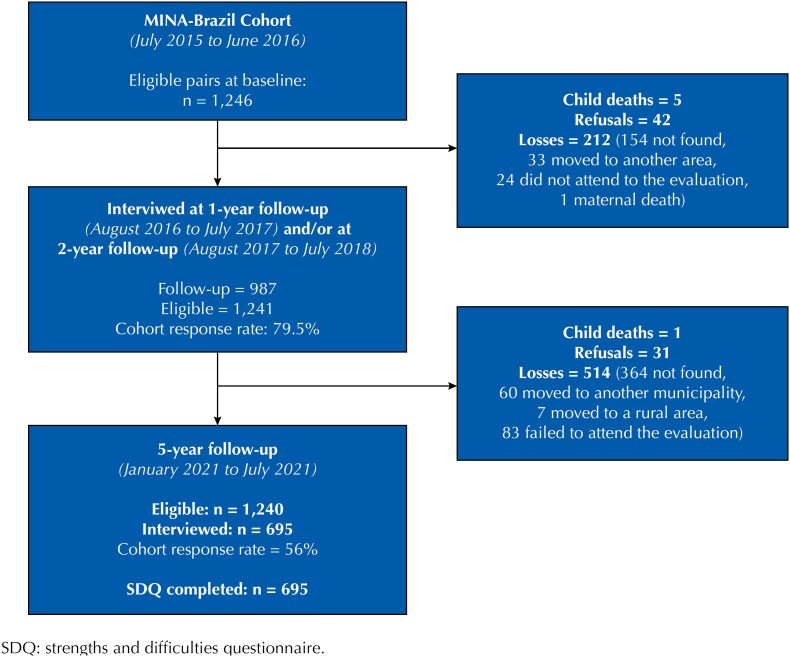
Flowchart of participants in the MINA-Brazil Birth Cohort Study for baseline and follow-up assessments.

At the five-year follow-up visit, conducted during covid-19 restrictions from 15 January to 4 July, 2021, 695 out of the 1,240 eligible children (56.0%) were enrolled^11^. Overall, less than 1% of the study children have reportedly attended childcare facilities since schools closed in early April 2020. All anthropometric measurements were conducted in duplicates using calibrated equipment and standardized procedures^16^. The children were barefoot, wore light clothes, and had no hairstyles. The stadiometers (to measure their height) were accurate to 0.1 millimeters and the scales (to measure the weight) had a 150 kg capacity and were accurate to 100 g (UM061, Tanita Corporation). The maximum allowed difference between measurements was 0.2 millimeters and 100 grams for height and weight, respectively. Z-scores were used according to the World Health Organization (WHO)^17^ to generate anthropometric indices. We adopted the WHO Anthroplus software (version 1.0.4) to assess the nutritional status of population groups using its nutritional survey module. Stunting was defined as a height-for-age z-score lower than −2 standard deviation (SD), thinness as a body mass index-for-age lower than −2 SD, overweight as a body mass index-for-age greater than +1 SD and lower than +2 SD, and obesity as a body mass index-for-age z-score greater than +2 SD.

Children participating in the 5-year follow-up visit had similar perinatal characteristics (sex, gestational age, preterm birth, and birth weight) to those lost at follow-up (n = 514). However, they differed significantly in the proportion of children from the poorest families and mothers with ≤ 9 years of schooling (36.2 *versus* 44.1% and 26.4 *versus* 47.5%, respectively). A p < 0.01 was obtained for both these factors (χ^2^ test).

The SDQ-P was included as part of the five-year follow-up. Trained interviewers completed the Portuguese version of the SDQ-P with parents or caregivers at the time of the interview. In total, 25 questions about the child's behavioral problems or strengths on a scale of 0–2 (0 = "Not True," 1 = "Somewhat True," and 2 = "Certainly True") were responded by parents/caregivers. According to their responses, a score from 0-2 was assigned accordingly. Each of the following five scales had five items: emotional symptoms, conduct problems, hyperactivity, peer problems, and prosocial behavior. The sum of the first four scales generated the total difficulty score (20 items). Overall, the higher the score, the greater the difficulty on the scale, except for prosocial behavior, which was reverse-scored (i.e., the higher the score, the better the prosocial behavior)^18^.

A similar procedure was adopted for the three-solution factorial model. Internalizing problems were considered the sum of emotional and peer symptoms, whereas externalizing problems, the sum of hyperactivity symptoms^5^.

### Statistical Analyses

Data were collected using tablets programmed with The Census and Survey Processing System (CSPro) 218 and transferred to Stata 17.0 (StataCorp, College Station, TX, USA) for statistical analysis. Supervisors routinely checked all information and provided feedback to research team members to correct inconsistencies whenever necessary.

Socioeconomic, obstetric, and perinatal characteristics included frequencies, means, and SD. Descriptive SDQ analysis for all children were conducted, who were divided by sex, including the mean score, SD, median, and interquartile intervals (25^th^ and 75^th^ percentiles).

The proportion of children was calculated according to the original instrument cut offs based on UK norms, classifying them as "close to average", "slightly raised", "high", and "very high" problems in each scale (except for the prosocial scale which can be classified as "close to average", "slightly lowered", "low", and "very low")^18^. Furthermore, cut-off points were estimated based on the SDQ distribution of our study sample. A similar methodology to that originally used by the authors of the instrument was followed based on a large UK population-based survey. Therefore, the estimated cut offs were elaborated to classify 80% of the children as having "close to average"; 10%, as "slightly raised"; 5%, as "high"; and 5%, as "very high" problems in each scale (except for the prosocial scale whose scoring is reverse, i.e., 80% were classified as "close to average"; 10%, as "slightly raised"; 5%, as "low"; and 5% as "very low").

The internal consistency of all scales was assessed using Cronbach's alpha coefficients and McDonald's omega (ω). A study conducted with the SDQ-P version and Dutch children aged from four to seven years suggested that McDonald's omega (ω) may be a more suitable alternative than Cronbach's alpha (α)^19^. However, to enable comparability with other studies, both coefficients are shown.

## RESULTS

Table 1 describes the sociodemographic, anthropometric, and perinatal characteristics of the 695 mother-child pairs at the five-year follow-up, in which children were aged between 58.5 to 71.9 months (average age 63.5 months, SD 2.71). Regarding nutritional status, only 2% of the children were classified as stunted and 1% as thin but 16% were overweight and 13% were obese, according to the WHO^17^ growth parameters. Overall, 49% of the children were girls and almost half of the families were *Bolsa Família* Program beneficiaries (45%). At birth, 7% were born with low weight (< 2,500 g) and 9% were premature; 50% of the deliveries were caesarean. Furthermore, the average maternal age was 25.7 years, with 21% of adolescents (< 19 years old). Most mothers self-declared non-white (88%), some lived in a different home than their partners (21%), had a low level of schooling (27% attended school for nine years or less), and were *Bolsa Família* Program beneficiaries (36%) at baseline.

**Table 1:** Participants' socioeconomic, anthropometric, and perinatal characteristics (n = 695).

Variables			Total	
			n^a^ or mean	% or SD
Five-year follow-up				
	Age (months)		63.5	2.7
	Sex			
	Girls		343	49.4
	Stunting^b^ (n = 692)		16	2.3
	Thinness^c^ (n = 692)		9	1.3
	Overweight^d^ (n = 692)		112	16.1
	Obesity^e^ (n = 692)		93	13.4
	Beneficiary of the *Bolsa Família* Program		312	44.9
Variables collected at birth				
	Birth weight (n = 694)			
		< 2,500 grams	51	7.3
		≥ 2,5000 grams	643	92.7
		Prematurity	63	9.1
	Type of birth			
		Normal	351	50.5
		Cesarean	344	49.5
		Maternal age (years)	25.7	6.7
	Self-reported maternal skin color (n = 680)			
		White	83	12.2
		Non-white	597	87.8
	Living with a partner (n = 680)			
		No	145	21.3
		Yes	535	78.7
	Maternal schooling (n = 679)			
		≤ 9 years	180	26.5
		10–12 years	348	51.3
		> 12 years	151	22.2
Beneficiary of the *Bolsa Família* Program			247	36.3

SD: standard deviation.

a Totals differ due to missing values.

b Stunting: height-for-age in z score <−2 standard deviation from the median of the World Health Organization (WHO)^17^.

c Thinness: body mass index-for-age in z score <−2 SD from the median of the WHO^17^.

d Overweight: body mass index-for-age in z score >+1 standard deviation and <+2 standard deviation from the median of the WHO^17^.

e Obesity: body mass index-for-age in z score >+2 standard deviation from the median of the WHO^17^.

Table 2 shows the distribution of "caseness" of SDQ data in the MINA-Brazil population according to the original score banding ("close to average", "slightly raised/lowered", "high/low", and "very high/very low"). This study observed frequency differences by comparing the proportion of children with behavior problems according to the original cut offs and those based on our study population distribution (Table 2). The behavior problem scale showed that the highest proportion of children was classified as having high or very high problems (UK norms, 25% *versus* MINA-Brazil, 11%); followed by emotional problems (UK norms 23% *versus* MINA, 7%); peer problems (UK norms 15% *versus* MINA, 7%); and hyperactivity (UK norms 13% *versus* MINA, 6%). Regarding prosocial behavior scale, 13% of children were classified as low or very low according to the UK norms and only 6% according to the MINA band. For the total difficulties scale, the proportion of children in the high or very high category totaled 10% when using the MINA banding, and almost twice (18%) when using the UK norms.

**Table 2:** SDQ classification at 5-6 years of age according to the original four-band categorization and the cut-off points based on the distribution of MINA Study data (n = 695).

Variables		Original^a^		MINA Brazil^b^	
		Cut off points	n (%)	Cut off points	n (%)
Emotional problems					
	Close to average	0–3	418 (60.1)	0–4	538 (77.4)
	Slightly raised	4	120 (17.3)	5–6	105 (15.1)
	High	5–6	105 (15.1)	7	19 (2.7)
	Very high	7–10	52 (7.5)	8–10	33 (4.8)
Conduct problems					
	Close to average	0–2	436 (62.7)	0–3	523 (75.2)
	Slightly raised	3	87 (12.5)	4–5	95 (13.7)
	High	4–5	95 (13.7)	6	46 (6.6)
	Very high	6–10	77 (11.1)	7–10	31 (4.5)
Hyperactivity					
	Close to average	0–5	465 (66.9)	0–6	552 (79.4)
	Slightly raised	6–7	143 (20.6)	7–8	99 (14.2)
	High	8	43 (6.2)	9	17 (2.5)
	Very high	9–10	44 (6.3)	10	27 (3.9)
Peer problems					
	Close to average	0–2	543 (78.1)	0–2	543 (78.1)
	Slightly raised	3	49 (7.1)	3–4	103 (14.8)
	High	4	54 (7.7)	5–6	34 (4.9)
	Very high	5–10	49 (7.1)	7–10	15 (2.2)
Prosocial behavior					
	Close to average	8–10	556 (80)	8–10	556 (80)
	Slightly lowered	7	50 (7.2)	6–7	98 (14.1)
	Low	6	48 (6.9)	5	18 (2.6)
	Very low	0–5	41 (5.9)	0–4	23 (3.3)
Total difficulties					
	Close to average	0–13	465 (66.9)	0–16	567 (81.6)
	Slightly raised	14–16	102 (14.7)	17–19	59 (8.5)
	High	17–19	59 (8.5)	20–22	35 (5)
	Very high	20–40	69 (9.9)	23–40	34 (4.9)

SDQ: strengths and difficulties questionnaire.

a Original cut offs: https://www.sdqinfo.org/.

b Cut offs according to Maternal and Child Health and Nutrition in Acre, Brazil percentiles (MINA-Brazil).

Table 3 shows the mean, SD, median and interquartile ranges, Cronbach's alpha, and McDonald's omega value for each scale considering the five-factor SDQ solution. Cronbach's alpha and McDonald's omega values indicated that the scales had moderate item internal consistency (0.60 ≤ α ≤ 0.40), except for the total difficulty score, which was considered substantial (α > 0.61). This research found similar results for McDonald's omega values, which ranged from 0.43-0.63.

**Table 3:** SDQ means, standard error, median, interquartile intervals, and internal consistency by sex considering the five-factor model (range of all scales 0-10, except ‘total difficulties,’ which ranges from 0 to 40).

Variable	All (n = 695)		Boys (n = 352)				Girls (n = 343)			
	mean (SD)	Cronbach's alpha / McDonald's omega	m (SD)	Median	p25–p75	Cronbach's alpha / McDonald's omega	m (SD)	Median	p25–p75	Cronbach's alpha / McDonald's omega
Emotional symptoms	3.1 (2.2)	0.44 / 0.47	3.0 (2.1)	3	2–4	0.40 / 0.42	3.1 (2.3)	3	1–4	0.48 / 0.51
Peer problems	1.6 (1.8)	0.40 / 0.43	1.6 (1.8)	1	0–2	0.42 / 0.44	1.6 (1.8)	1	0–2	0.38 / 0.43
Conduct problems	2.3 (2.2)	0.57 / 0.58	2.5 (2.3)	2	1–4	0.60 / 0.61	2.1 (2.0)	2	0–3	0.52 / 0.52
Hyperactivity	4.3 (2.6)	0.55 / 0.55	4.7 (2.6)	4	3–7	0.56 / 0.57	4.0 (2.5)	4	2–6	0.52 / 0.52
Prosocial behavior	8.6 (1.7)	0.45 / 0.47	8.3 (1.8)	9	7–10	0.45 / 0.47	8.9 (1.5)	10	8–10	0.42 / 0.43
Total difficulties	11.3 (6.1)	0.63 / 0.63	11.8 (6.2)	11	7–15	0.65 / 0.66	10.8 (5.8)	10	6–15	0.60 / 0.61

SDQ: strengths and difficulties questionnaire; SD: standard deviation.

Table 4 shows the mean, SD, median and interquartile ranges, Cronbach's alpha, and McDonald's omega values according to sex for each scale considering the three-factor SDQ solution. Cronbach's alpha showed that the internal consistency of the items was substantial for the externalizing problems scale (α = 0.69, ω = 0.70), and moderate for internalizing problems scale (α = 0.53, ω = 0.54).

**Table 4:** SDQ means, standard error, median, interquartile intervals, and internal consistency by sexconsidering the three-factor model (The externalizing score ranges from 0 to 20 and is the sum of the conduct and hyperactivity scales. The internalizing score ranges from 0 to 20 and is the sum of the emotional and peer problems scales. The prosocial behavior scale ranges from 0 to 10 and the ‘difficulties total’ ranges from 0 to 40).

Variable	All (n = 695)		Boys (n = 352)				Girls (n = 343)			
	m (SD)	Cronbach's alpha/ McDonald's omega	m (SD)	Median	p25–p75	Cronbach's alpha/ McDonald's omega	m (SD)	Median	p25–p75	Cronbach's alpha/ McDonald's omega
Internalizing problems	4.7 (3.2)	0.53 / 0.54	4.6 (3.1)	4	2–6	0.52 / 0.53	4.8 (3.2)	4	2–7	0.54 / 0.56
Externalizing problems	6.6 (4.1)	0.69 / 0.70	7.1 (4.2)	7	4–10	0.71 / 0.72	6.0 (3.9)	6	3–8	0.69 / 0.67
Prosocial behavior	8.6 (1.7)	0.45 / 0.47	8.3 (1.8)	9	7–10	0.45 / 0.47	8.9 (1.5)	10	8–10	0.42 / 0.43

SDQ: Strengths and Difficulties Questionnaire; SD: standard deviation.

Among the four scales, which comprised the total difficulties score, the hyperactivity scale had the highest mean, whereas the problems with peers scale had the lowest. Boys had higher means for the externalizing (mean: boys = 6.59, girls = 6.03), conduct (mean: boys = 2.48, girls = 2.08), and hyperactivity problems (mean: boys = 4.67, girls = 3.95) scales. Additionally, boys had a lower mean prosocial behavior (mean: boys = 8.32, girls = 8.89). Internalizing problems showed no differences between sexes.

## DISCUSSION

Using the five-factor structure and original SDQ cut offs, we found that 25% of the children were considered as having high or very high problems as per the conduct scale, 23% as per the emotional scale, 15% as per the peer problems scale, 13% as per the hyperactivity scale, and 13% (low or very low) as per the prosocial behavior scale. The total difficulties scale considered 18% of the children as having high or very high problems. However, numbers greatly differed from the percentages when using cut offs based on study sample percentiles. Furthermore, our results showed that the SDQ scales had modest or substantial internal consistency in Amazonian pre-school children.

As expected, our results differed from studies conducted with children in other countries using the same cut off values. A Chinese study with 950 pre-schoolers aged 3-5 years found that 39% of participants had emotional problems; 27%, conduct problems; and 23%, hyperactivity problems^20^, all higher than our results. However, a study with 800 Pakistani children aged 6-16 years showed that 23% of their sample had emotional problems; 27%, conduct problems; 11%, hyperactivity; 13%, peer problems; 3% low or very low prosocial behavior; and 16%, total behavioral difficulties^21^.

Overall, when comparing our results with those of other studies conducted in Brazil before the covid-19 pandemic, MINA-Brazil cohort participants showed greater behavioral disorders. Data from a Brazilian south cohort with 3,718 children showed that, at six years of age, 14.7 (16.1% boys; 13.1% girls) and 13.5% (13.0% boys; 14.0% girls) had high or very high levels of conduct and emotional problems, respectively^22^. Another study conducted in southern Brazil with 152 school children aged 6-13 years observed higher behavior problems in children living near recycling sites exposed to urban waste than to those living more than 150 meters away^23^. In that study, the authors defined ‘caseness’ as children on the borderline and abnormal problems categories (equivalent to the sum of "slightly raised," "high," and "very high" classifications in this study). Caseness frequency differed between children for total difficulties (24% in the exposed group vs. 10% in the control group) and was lower than MINA-Brazil data, which was 33.1% based on the same cut-offs. However, for some of the other SDQ subscales, our results were lower than those for the control group (i.e., hyperactivity and peer problems subscales).

When using a broader internalizing and externalizing model, we also observed differences between our results and those of other Brazilian studies before the covid-19 pandemic. Data from a cross-sectional study in southeastern Brazil with 825 children aged six to 11 years showed a higher frequency of internalizing problems (30.7%) than externalizing problems (18.3%)^24^. This contrasted with our results (16 and 17% for internalizing and externalizing problems, respectively, in the MINA-Brazil sample).

In this study, the SDQ was notably applied during the covid-19 pandemic, which has directly or indirectly caused unprecedented harm to the entire population, but especially to early childhood as it represents a vulnerable period in people's lives. Although children are not considered a group risk for SARS-CoV-2 infection, the pandemic worsened compromising factors for child development, such as parental stress, suspension of school activities, social isolation, lack of physical activity, and food insecurity, particularly in unstructured contexts^25^. The covid-19 crisis in Brazil has been most dramatic across the Amazon, in which public health facilities were already operating near full capacity before the pandemic^11^. An Italian study with 463 children aged 5 to 17 years showed that confinement measures and changes in daily routine during the covid-19 pandemic negatively affected parents' psychological dimensions, thus exposing children to a significant risk to their well-being^26^. This may explain the higher frequency of behavior problems in our study than expected according to other national and international studies.

We observed frequency differences when comparing the proportion of children with behavioral problems according to the original SDQ cut offs and those elaborated in this study based on our population distribution. In agreement, recent studies have suggested that using European norms in different contexts may fail to reflect local realities. Data from different continents support that, for clinical purposes, ‘caseness’ should be defined by in-country cut offs^4^.

Based on eight population-based studies from seven countries (Bangladesh, Brazil, Britain, India, Norway, Russia, and Yemen) with data from more than 29,225 children aged 5-16 years, the authors concluded that SDQ cross-country differences fail to necessarily reflect comparable differences in disorder rates^27^. Therefore, interpreting frequencies and other results from cross-cultural comparisons of mental health requires caution, especially when assessments rely on brief questionnaires such as the SDQ^27^.

To investigate the measure of a construct by multiple items, many studies on social and behavioral problems have adopted Cronbach's alpha, including those using the SDQ-P. However, Cronbach's alpha may underestimate the true reliability of a construct. Therefore, McDonald's omega has been increasingly adopted and reported^28^. Although many SDQ studies have found no significant differences between the two coefficients, some authors recommend using McDonald's omega^19^. Our results concurred with previous studies. We observed higher McDonald omega coefficients than Cronbach's alpha coefficients for most SDQ scales, including when stratified by sex.

Our internal consistency analysis supported the potential usefulness of SDQ in the setting of this study. Results showed moderate internal reliability for all scales (0.60 ≤ α ≤ 0.40), except for total difficulty scores, which was substantial (α > 0.61). A study on Brazilian children aged 6 and 11 years found similar results: Cronbach's alphas ranged from 0.59-0.65 and 0.52-0.59 for conduct and emotional problems subscales, respectively^22^.

In concurrence with other studies, our analysis showed that the peer problems scale had the worst internal consistency. A review of 26 studies showed that the peer problems scale items might fail to reflect the same construct as its alphas were the lowest for the SDQ^29^.

### Strengths and Limitations

This study makes significant contributions to mental health assessments in early childhood in the Amazon during the covid-19 restrictions. It helps to comprehend the limitations and potential of the SDQ, a low-cost and easily applicable screening tool. It is worth mentioning that since data about behavior problems during childhood in the Brazilian north are rare, comparisons require careful interpretations. We also analyzed data from a large population-based birth cohort using validated instruments that attributed credibility to our findings. However, some important limitations of this study require consideration. We estimated SDQ-P cut offs based on the distribution of our own study population, which includes a narrow age range (58.5 to 71.9 months) and has very unique socioeconomic and cultural features. Thus, these results should not be generalized to other populations. Also, although our internal consistency analyses supports the usefulness of SDQ in our population, we recognize the existence of different possible analysis to explore other important psychometric characteristics of an instrument^30^, which was not the focus of this study. Moreover, from the original MINA-Brazil participants enrolled at birth (n =1,240), the five-year assessment had a 56% retention rate (n = 695). Families of children lost to follow-up had a lower socioeconomic status and maternal education than those who attended the five-year follow-up. Thus, as the literature considers that a socially vulnerable background correlates with poor child development, the frequency of behavior disorders reported in this study could be even higher.

## CONCLUSION

Our results regarding child behavior problems reinforce the need for mental health care in early life in the Amazon region. Furthermore, our internal consistency results agreed with those of other national studies, supporting the usefulness of the SDQ. However, interpreting our results and comparing them with those of other studies requires caution due to the cultural and contextual diversity across different settings.
