# Supplementary material for: Internal consistency of the Strengths and Difficulties Questionnaire in Amazonian children
Source: Rev Saude Publica. 2024 Feb 1;57(Suppl 2):4s. doi: 10.11606/s1518-8787.2023057005562 (PMC10897965; doi:10.11606/s1518-8787.2023057005562)
Supplement: Translation

# Translation: Consistência interna do Questionário de Capacidades e Dificuldades em crianças amazônicas

**Authors:** Isabel Giacomini, Maria Rosário O. Martins, Alicia Matijasevich, Marly A. Cardoso

**Affiliations:** I São Paulo SP Brasil Universidade de São Paulo. Faculdade de Saúde Pública. Programa de Pós-Graduação em Nutrição em Saúde Pública. São Paulo, SP, Brasil; II Lisboa Portugal Universidade NOVA de Lisboa. Instituto de Higiene e Medicina Tropical, Saúde Global e Medicina Tropical. Lisboa, Portugal; III São Paulo SP Brasil Universidade de São Paulo. Faculdade de Medicina. Departamento de Medicina Preventiva. São Paulo, SP, Brasil; IV São Paulo SP Brasil Universidade de São Paulo. Faculdade de Saúde Pública. Departamento de Nutrição. São Paulo, SP, Brasil

**Keywords:** Psicometria, Comportamento Problema, Transtornos do Comportamento Infantil, Escala de Avaliação Comportamental

## Abstract

**OBJETIVO:**

Descrever a frequência de problemas de comportamento e a consistência interna da versão para os pais do Questionário de Capacidades e Dificuldades (SDQ-P) em crianças pré-escolares da Amazônia durante a pandemia de covid-19.

**MÉTODOS:**

Foram utilizados dados do estudo de saúde e nutrição Materno-Infantil no Acre (MINA-Brasil), uma coorte de nascimentos de base populacional na Amazônia Ocidental Brasileira. O SDQ-P foi aplicado aos pais e cuidadores em 2021 na visita de acompanhamento de cinco anos de 695 crianças (49,4% das quais eram meninas). Esse instrumento é um breve questionário de rastreamento comportamental composto por 25 itens reorganizados em cinco subescalas: sintomas emocionais, problemas de conduta, hiperatividade/desatenção, problemas de relacionamento com colegas e comportamento pró-social. Os casos de problemas de comportamento foram definidos de acordo com os pontos de corte originais do SDQ, baseados nas normas do Reino Unido. Além disso, os pontos de corte foram estimados com base nos percentis dos resultados do SDQ-P da amostra do nosso estudo. A consistência interna foi avaliada pelo cálculo do coeficiente alfa de Cronbach e ômega de McDonald para cada escala.

**RESULTADOS:**

De acordo com os pontos de corte baseados na distribuição da população estudada, 10% de todas as crianças apresentaram escores totais de dificuldade elevados ou muito elevados, o que quase dobrou quando os pontos de corte originais do SDQ, baseados nas normas do Reino Unido, foram utilizados (18%). Este estudo também encontrou diferenças nas demais escalas. Comparados às meninas, os meninos apresentaram maiores médias de problemas de externalização e menores médias de comportamento pró-social. O modelo de cinco fatores apresentou consistência interna dos itens moderada para todas as escalas (0,60 ≤ α ≤ 0,40), exceto para a escala de pontuação total de dificuldades, a qual foi considerada substancial (α > 0,61).

**CONCLUSÕES:**

Nossos resultados apoiam a utilidade do SDQ em nossa população de estudo e reforçam a necessidade de estratégias e desenvolvimento de políticas para o cuidado em saúde mental no início da vida na Amazônia.

## INTRODUÇÃO

Estimativas sugerem que cerca de 20% de todas as crianças no mundo com menos de sete anos de idade experimentam algum tipo de transtorno mental^1^. Assim, abordar a saúde mental e o desenvolvimento psicossocial precoce da criança diz respeito tanto à sua chance de alcançar uma vida plena quanto à formação de capital humano. A primeira infância representa um período particularmente crítico e vulnerável, pois as crianças em seus primeiros anos de vida respondem a traumas e estresse ou expressam suas emoções e sentimentos de maneiras específicas, diferentes daquelas das crianças mais velhas^1,2^.

A frequência de transtornos mentais na infância varia entre os países. Dados de crianças com idade entre 36 e 59 meses de 63 países mostraram que a frequência de suspeita de atraso socioemocional é heterogênea, variando de 20% em países de alta renda a 18%, 25% e 32% em países de renda média alta, média baixa e baixa, respectivamente^2^.

O Questionário de Capacidades e Dificuldades (SDQ) tem como objetivo rastrear problemas de comportamento em crianças de dois a 17 anos^3^. Está disponível em mais de 40 idiomas e em três versões: para professores (SDQ-T), pais/responsáveis (SDQ-P) e autorrelato (SDQ-S). Ele inclui 25 questões sobre comportamentos ou habilidades das crianças que podem ser organizadas em cinco escalas: sintomas emocionais, problemas de conduta, hiperatividade, problemas com colegas e comportamento pró-social.

Os resultados do SDQ podem ser explorados como escores brutos ou em categorias, utilizando os pontos de corte propostos pelos autores do instrumento. As faixas de categorização foram estabelecidas de acordo com um grande estudo de base populacional no Reino Unido (UK), no qual 80% das crianças foram classificadas como "próximas da média"; 10%, como "ligeiramente elevado"; 5%, como "elevado" e 5%, como "muito elevado" em relação a problemas de comportamento em cada escala (exceto para a escala pró-social, que é pontuada de forma inversa). No entanto, considerando as diferenças socioeconômicas e culturais entre os países, estudos fora da Europa sugerem que faixas baseadas em cada distribuição amostral deveriam ser consideradas para comparações^4^.

Mais recentemente, como alternativa ao modelo de cinco escalas já mencionado, uma nova solução de três fatores foi proposta para amostras de baixo risco ou da população geral^5^. Esse modelo reorganiza os itens do SDQ em três subescalas: problemas internalizantes (soma dos sintomas emocionais e de colegas), problemas externalizantes (soma dos sintomas de conduta e hiperatividade) e comportamento pró-social^5^.

O SDQ foi validado no Brasil por meio de uma avaliação em duas fases em diferentes contextos locais para avaliar sua confiabilidade teste-reteste^6–8^. Sua implementação é fácil e quando os usuários recebem treinamento prévio, não há necessidade de profissionais especializados^8^. Além disso, considerando a utilização extensa do SDQ pela comunidade científica, os resultados podem ser comparados com estudos nacionais e internacionais^9,10^.

Embora o SDQ tenha sido aplicado em diferentes locais no Brasil, ele não tem sido utilizado em áreas remotas do país e sem avaliação de sua consistência interna. Na Amazônia brasileira, as crianças das famílias socioeconomicamente mais vulneráveis são desproporcionalmente afetadas pelas infecções por SARS-CoV-2^11^. As grandes desigualdades sociais e vulnerabilidades exigem dados sobre o comportamento de crianças na primeira infância na Amazônia. Portanto, este estudo descreve a frequência de distúrbios de comportamento em crianças pré-escolares durante as restrições covid-19. O presente estudo fez essa avaliação por meio das escalas do SDQ-P de acordo com sua categorização quádrupla original e analisou a consistência interna dos modelos de cinco e três soluções propostos por Goodman^3^ e Goodman et al.^5^, respectivamente, em uma coorte de nascimentos na Amazônia Ocidental Brasileira.

## MÉTODOS

### Desenho, local e população do estudo

O estudo de saúde e nutrição Materno-Infantil no Acre (Estudo MINA-Brasil) é uma coorte de nascimentos de base populacional realizada no município de Cruzeiro do Sul, Estado do Acre, Amazônia Ocidental Brasileira. Em 2021, Cruzeiro do Sul possuía uma população estimada em 89.760 indivíduos (cerca de 70% dos quais residiam em áreas urbanas)^12^. O município representa o segundo maior produto interno bruto municipal do Estado do Acre e possui um Índice de Desenvolvimento Humano de 0,664^12^, abaixo da média nacional^13^.

Este estudo foi aprovado pelo Comitê de Ética em Pesquisa da Faculdade de Saúde Pública da Universidade de São Paulo (# 872.613, 2014; # 2.358.129, 2017). O termo de consentimento livre e esclarecido foi obtido de todas as mães participantes ou dos pais ou responsáveis pelas crianças (se as mães tivessem idade < 18 anos).

### Avaliações na linha de base e de acompanhamento

Em 2015, os pares mãe-bebê foram incluídos no estudo em clínicas públicas de pré-natal ou no momento do parto no Hospital da Mulher e da Criança do Vale do Juruá, única maternidade de Cruzeiro do Sul, conforme descrito anteriormente^14^. Na linha de base, 1.246 pares mãe-bebê eram elegíveis para consultas de acompanhamento. No nascimento, foram obtidas informações sociodemográficas maternas, como cor da pele autorreferida (branca, parda, amarela, preta ou ameríndia); situação conjugal (mora com companheiro ou não); escolaridade (anos de estudo); e condição de beneficiário do *Programa Bolsa Família* programa de transferência condicionada de renda direcionado a famílias em situação de pobreza (sim ou não)^15^. Dados obstétricos e perinatais (peso ao nascer – em gramas, idade gestacional e tipo de parto) foram coletados dos registros da maternidade. Após a avaliação na linha de base, foram realizadas visitas de acompanhamento nas idades de um, dois e cinco anos (Figura).

**Figura 1:** Fluxograma das participantes do Estudo de Coorte de Nascimentos MINA-Brasil para avaliação basal e de acompanhamento.

Na visita de acompanhamento de cinco anos, realizada durante as restrições covid-19 de 15 de janeiro a 4 de julho de 2021, 695 das 1.240 crianças elegíveis (56,0%) foram avaliadas^11^. No geral, menos de 1% das crianças do estudo frequentaram creches desde que as escolas fecharam no início de abril de 2020. Todas as medidas antropométricas foram realizadas em duplicata, utilizando equipamentos calibrados e procedimentos padronizados^16^. As crianças estavam descalças, usavam roupas leves e sem penteados. Os estadiômetros (para aferir a altura) tinham precisão de 0,1 mm e a balança (para aferir o peso) tinha capacidade de 150 kg e precisão de 100 g (UM061, Tanita Corporation). A diferença máxima permitida entre as medidas foi de 0,2 milímetros e 100 gramas para altura e peso, respectivamente. Os escores z foram utilizados de acordo com a Organização Mundial da Saúde (OMS)^17^ para gerar índices antropométricos. Adotou-se o software WHO Anthroplus (versão 1.0.4) para avaliar o estado nutricional de grupos populacionais utilizando seu módulo de inquérito nutricional. O déficit de estatura foi definido como escore z de estatura para idade menor que −2 desvio padrão (DP), magreza como índice de massa corporal para idade menor que −2 DP, sobrepeso como índice de massa corporal para idade maior que +1 DP e menor que +2 DP e obesidade como escore z de índice de massa corporal por idade maior que +2 DP.

As crianças que participaram da consulta de acompanhamento de cinco anos apresentaram características perinatais (sexo, idade gestacional, prematuridade e peso ao nascer) semelhantes àquelas que não realizaram o acompanhamento (n = 514). No entanto, diferiram significativamente na proporção de crianças das famílias mais pobres e de mães com ≤ 9 anos de estudo (36,2 versus 44,1% e 26,4 versus 47,5%, respectivamente). Obteve-se um p < 0,01 para ambos os fatores (teste qui-quadrado).

O SDQ-P foi incluído como parte do acompanhamento de cinco anos. Entrevistadores treinados aplicaram a versão em português do SDQ-P aos pais ou cuidadores no momento da entrevista. No total, 25 questões sobre os problemas ou capacidades de comportamento da criança em uma escala de 0 a 2 (0 = "Não Verdadeiro", 1 = "Levemente Verdadeiro" e 2 = "Certamente Verdadeiro") foram respondidas pelos pais/cuidadores. De acordo com suas respostas, uma pontuação de 0–2 foi atribuída. Cada uma das cinco escalas continha cinco itens: sintomas emocionais, problemas de conduta, hiperatividade, problemas com colegas e comportamento pró-social. A soma das quatro primeiras escalas gerou a pontuação total de dificuldades (20 itens). No geral, quanto maior o escore, maior a dificuldade na escala, com exceção do comportamento pró-social, que foi pontuado de forma reversa (ou seja, quanto maior o escore, melhor o comportamento pró-social)^18^.

Um procedimento semelhante foi adotado para o modelo fatorial de três soluções. Problemas internalizantes foram considerados como a soma dos sintomas emocionais e dos colegas, enquanto os problemas externalizantes, a soma dos sintomas de hiperatividade^5^.

### Análises Estatísticas

Os dados foram coletados em *tablets* programados com o *Census and Survey Processing System* (CSPro) 218 e transferidos para o Stata 17.0 (StataCorp, College Station, TX, EUA) para análise estatística. Os supervisores verificavam rotineiramente todas as informações e davam retorno aos membros da equipe de pesquisa para corrigir inconsistências sempre que necessário.

As características socioeconômicas, obstétricas e perinatais incluíram frequências, médias e DP. Foi realizada análise descritiva do SDQ para todas as crianças, que foram divididas por sexo, incluindo o escore médio, DP, mediana e intervalos interquartis (percentis 25º e 75°).

A proporção de crianças foi calculada de acordo com os pontos de corte do instrumento original com base nas normas do Reino Unido, classificando-as em problemas "próximos da média", "levemente elevados", "elevados" e "muito elevados" em cada escala (exceto para a escala pró-social, que pode ser classificada como "próxima da média", "ligeiramente baixo", "baixo" e "muito baixo")^18^. Além disso, os pontos de corte foram estimados com base na distribuição do SDQ da amostra do nosso estudo. Uma metodologia semelhante à originalmente utilizada pelos autores do instrumento foi utilizada de forma análoga a uma pesquisa com grande amostra de base populacional no Reino Unido. Assim, os pontos de corte estimados foram elaborados para classificar 80% das crianças como tendo problemas "próximos da média"; 10%, como "ligeiramente elevados"; 5%, como "elevados"; e 5%, como "muito elevados" em cada escala (exceto na escala pró-social, cuja pontuação é inversa, ou seja, 80% foram classificados como "próximos da média"; 10%, como "ligeiramente abaixo"; 5%, como "baixo" e 5% como "muito baixo").

A consistência interna de todas as escalas foi avaliada por meio dos coeficientes alfa de Cronbach e ômega de McDonald (ω). Um estudo realizado com a versão SDQ-P e crianças holandesas de quatro a sete anos sugeriu que o ômega de McDonald (ω) pode ser uma alternativa mais adequada do que o alfa de Cronbach (α)^19^. No entanto, para permitir a comparabilidade com outros estudos, ambos os coeficientes são apresentados.

## RESULTADOS

A Tabela 1 descreve as características sociodemográficas, antropométricas e perinatais dos 695 pares mãe-filho no acompanhamento de cinco anos, no qual as crianças tinham entre 58,5 e 71,9 meses (média de idade de 63,5 meses, DP 2,71). Em relação ao estado nutricional, apenas 2% das crianças foram classificadas como de baixa estatura e 1% como magras, mas 16% apresentavam sobrepeso e 13% eram obesas, de acordo com os parâmetros de crescimento da OMS^17^. No geral, 49% das crianças eram meninas e quase metade das famílias eram beneficiárias do *Programa Bolsa Família* (45%). No nascimento, 7% nasceram com baixo peso (< 2.500 g) e 9% prematuras; 50% dos partos foram cesáreas. Além disso, a média de idade materna foi de 25,7 anos, com 21% de adolescentes (< 19 anos). A maioria das mães autodeclararam-se não brancas (88%), algumas moravam em domicílio diferente do companheiro (21%), tinham baixa escolaridade (27% frequentaram a escola por nove anos ou menos) e eram beneficiárias do *Programa Bolsa Família* (36%) na linha de base.

**Tabela 1:** Características socioeconômicas, antropométricas e perinatais dos participantes (n = 695).

Variáveis			Total	
			n^a^ ou média	% ou DP
Acompanhamento de cinco anos				
	Idade (meses)		63,5	2,7
	Sexo			
	Meninas		343	49,4
	Déficit de estatura^b^ (n = 692)		16	2,3
	Magreza^c^ (n = 692)		9	1,3
	Sobrepeso^d^ (n = 692)		112	16,1
	Obesidade^e^ (n = 692)		93	13,4
	Beneficiário do *Programa Bolsa Família*		312	44,9
Variáveis coletadas no nascimento				
	Peso ao nascer (n = 694)			
		< 2.500 gramas	51	7,3
		≥ 2.500 gramas	643	92,7
		Prematuridade	63	9,1
	Tipo de parto			
		Normal	351	50,5
		Cesariana	344	49,5
		Idade materna (anos)	25,7	6,7
	Cor da pele materna autorreferida (n = 680)			
		Branca	83	12,2
		Não-branca	597	87,8
	Mora com companheiro (n = 680)			
		Não	145	21,3
		Sim	535	78,7
	Escolaridade materna (n = 679)			
		≤ 9 anos	180	26,5
		10–12 anos	348	51,3
		> 12 anos	151	22,2
Beneficiário do *Programa Bolsa Família*			247	36,3

DP: desvio padrão.

a Os totais diferem devido a valores ausentes.

b Déficit de estatura: estatura/idade em escore z < −2 DP da mediana da Organização Mundial da Saúde (OMS)^17^.

c Magreza: índice de massa corporal para idade em escore z < −2 desvio padrão da mediana da OMS^17^.

d Sobrepeso: índice de massa corporal para idade em escore z > +1 desvio padrão e < +2 desvio padrão da mediana da OMS^17^.

e Obesidade: índice de massa corporal para idade em escore z > +2 desvio padrão da mediana da OMS^17^.

A Tabela 2 mostra a distribuição da frequência de casos de problemas de comportamento de acordo com os resultados do SDQ na população do MINA-Brasil utilizando os pontos de corte originais ("próximo da média", "levemente elevado/baixo", "elevado/baixo" e "muito elevado/muito baixo"). O presente estudo observou diferenças de frequência comparando a proporção de crianças com problemas de comportamento de acordo com os pontos de corte originais e aqueles baseados na distribuição da população do nosso estudo (Tabela 2). A escala de problemas de conduta apresentou a maior proporção de crianças com problemas elevados ou muito elevados (normas do Reino Unido, 25%, versus MINA-Brasil, 11%), seguida por problemas emocionais (normas do Reino Unido, 23%, versus MINA, 7%), problemas com colegas (normas do Reino Unido, 15%, versus MINA, 7%) e hiperatividade (normas do Reino Unido, 13%, versus MINA, 6%). Em relação à escala de comportamento pró-social, 13% das crianças foram classificadas como baixo ou muito baixo de acordo com as normas do Reino Unido e apenas 6% de acordo com a faixa MINA. Para a escala total de dificuldades, a proporção de crianças na categoria elevada ou muito elevada totalizou 10% usando a faixa da MINA-Brasil – quase o dobro (18%) quando as normas do Reino Unido foram utilizadas.

**Tabela 2:** Classificação do SDQ dos cinco a seis anos de idade de acordo com a categorização original de quatro bandas e os pontos de corte baseados na distribuição dos dados do Estudo MINA (n = 695).

Variáveis		Original^a^		MINA Brasil^b^	
		Pontos de corte	n (%)	Pontos de corte	n (%)
Problemas emocionais					
	Perto da média	0–3	418 (60,1)	0–4	538 (77,4)
	Levemente elevado	4	120 (17,3)	5–6	105 (15,1)
	Elevado	5–6	105 (15,1)	7	19 (2,7)
	Muito elevado	7–10	52 (7,5)	8–10	33 (4,8)
Problemas de conduta					
	Perto da média	0–2	436 (62,7)	0–3	523 (75,2)
	Levemente elevado	3	87 (12,5)	4–5	95 (13,7)
	Elevado	4–5	95 (13,7)	6	46 (6,6)
	Muito elevado	6–10	77 (11,1)	7–10	31 (4,5)
Hiperatividade					
	Perto da média	0–5	465 (66,9)	0–6	552 (79,4)
	Levemente elevado	6–7	143 (20,6)	7–8	99 (14,2)
	Elevado	8	43 (6,2)	9	17 (2,5)
	Muito elevado	9–10	44 (6,3)	10	27 (3,9)
Problemas com colegas					
	Perto da média	0–2	543 (78,1)	0–2	543 (78,1)
	Levemente elevado	3	49 (7,1)	3–4	103 (14,8)
	Elevado	4	54 (7,7)	5–6	34 (4,9)
	Muito elevado	5–10	49 (7,1)	7–10	15 (2,2)
Comportamento pró-social					
	Perto da média	8–10	556 (80)	8–10	556 (80)
	Ligeiramente baixo	7	50 (7,2)	6–7	98 (14,1)
	Baixo	6	48 (6,9)	5	18 (2,6)
	Muito baixo	0–5	41 (5,9)	0–4	23 (3,3)
Total de dificuldades					
	Perto da média	0–13	465 (66,9)	0–16	567 (81,6)
	Levemente elevado	14–16	102 (14,7)	17–19	59 (8,5)
	Elevado	17–19	59 (8,5)	20–22	35 (5)
	Muito elevado	20–40	69 (9,9)	23–40	34 (4,9)

SDQ: questionário de capacidades e dificuldades.

a Pontos de corte originais: https://www.sdqinfo.org/.

b Pontos de corte segundo percentis da coorte saúde e nutrição Materno-Infantil no Acre (MINA-Brasil).

A Tabela 3 mostra a média, DP, mediana e intervalos interquartis, alfa de Cronbach e ômega de McDonald para cada escala considerando a solução SDQ de cinco fatores. Valores de alfa de Cronbach e ômega de McDonald indicaram que as escalas apresentaram consistência interna dos itens moderada (0,60 ≤ α ≤ 0,40), com exceção da escala total de dificuldades que foi considerado substancial (α > 0,61). Resultados semelhantes foram encontrados para os valores de ômega de McDonald, que variaram entre 0,43 e 0,63.

**Tabela 3:** Médias do SDQ, desvio padrão, mediana, intervalos interquartis e consistência interna por sexo, considerando o modelo de cinco fatores (variação de todas as escalas de 0 a 10, exceto "total de dificuldades", que varia de 0 a 40).

Variável	Todos (n = 695)		Meninos (n = 352)				Meninas (n = 343)			
	m (SD)	Alfa de Cronbach / Ômega de McDonald	m (SD)	Mediana	p25–p75	Alfa de Cronbach / Ômega de McDonald	m (SD)	Mediana	p25–p75	Alfa de Cronbach / Ômega de McDonald
Sintomas emocionais	3,1 (2,2)	0,44 / 0,47	3,0 (2,1)	3	2–4	0,40 / 0,42	3,1 (2,3)	3	1–4	0,48 / 0,51
Problemas com colegas	1,6 (1,8)	0,40 / 0,43	1,6 (1,8)	1	0–2	0,42 / 0,44	1,6 (1,8)	1	0–2	0,38 / 0,43
Problemas de conduta	2,3 (2,2)	0,57 / 0,58	2,5 (2,3)	2	1–4	0,60 / 0,61	2,1 (2,0)	2	0–3	0,52 / 0,52
Hiperatividade	4,3 (2,6)	0,55 / 0,55	4,7 (2,6)	4	3–7	0,56 / 0,57	4,0 (2,5)	4	2–6	0,52 / 0,52
Comportamento pró-social	8,6 (1,7)	0,45 / 0,47	8,3 (1,8)	9	7–10	0,45 / 0,47	8,9 (1,5)	10	8–10	0,42 / 0,43
Total de dificuldades	11,3 (6,1)	0,63 / 0,63	11,8 (6,2)	11	7–15	0,65 / 0,66	10,8 (5,8)	10	6–15	0,60 / 0,61

SDQ: questionário de capacidades e dificuldades; DP: desvio padrão.

A Tabela 4 mostra os valores de média, DP, mediana e intervalo interquartil, alfa de Cronbach e ômega de McDonald de acordo com o sexo para cada escala considerando a solução SDQ de três fatores. O alfa de Cronbach mostrou que a consistência interna dos itens foi substancial para a escala de problemas externalizantes (α = 0,69, ω = 0,70) e moderada para a escala de problemas internalizantes (α = 0,53, ω = 0,54).

**Tabela 4:** Médias do SDQ, desvio padrão, mediana, intervalos interquartis e consistência interna por sexo, considerando o modelo de três fatores (o escore externalizante varia de 0 a 20 e é a soma das escalas de conduta e hiperatividade. O escore internalizante varia de 0 a 20 e é a soma das escalas de problemas emocionais e de problemas com colegas. A escala de comportamento pró-social varia de 0 a 10 e o total de dificuldades varia de 0 a 40).

Variável	Todos (n = 695)		Meninos (n = 352)				Meninas (n = 343)			
	m (SD)	Alfa de Cronbach / Ômega de McDonald	m (SD)	Mediana	p25–p75	Alfa de Cronbach / Ômega de McDonald	m (SD)	Mediana	p25–p75	Alfa de Cronbach / Ômega de McDonald
Problemas internalizantes	4,7 (3,2)	0,53 / 0,54	4,6 (3,1)	4	2–6	0,52 / 0,53	4,8 (3,2)	4	2–7	0,54 / 0,56
Problemas externalizantes	6,6 (4,1)	0,69 / 0,70	7,1 (4,2)	7	4–10	0,71 / 0,72	6,0 (3,9)	6	3–8	0,69 / 0,67
Comportamento pró-social	8,6 (1,7)	0,45 / 0,47	8,3 (1,8)	9	7–10	0,45 / 0,47	8,9 (1,5)	10	8–10	0,42 / 0,43

SDQ: questionário de capacidades e dificuldades; DP: desvio padrão.

Dentre as quatro escalas que compuseram a escala total de dificuldades, a escala de hiperatividade apresentou a maior média, enquanto a escala de problemas com colegas apresentou a menor média. Os meninos apresentaram maiores médias para as escalas de problemas externalizantes (média: meninos = 6,59, meninas = 6,03), de conduta (média: meninos = 2,48, meninas = 2,08) e de hiperatividade (média: meninos = 4,67, meninas = 3,95). Além disso, os meninos apresentaram menor média de comportamento pró-social (média: meninos = 8,32, meninas = 8,89). Não houve diferenças entre os sexos para os problemas internalizantes de comportamento.

## DISCUSSÃO

Utilizando a estrutura de cinco fatores e os pontos de corte originais do SDQ, verificou-se que 25% das crianças foram consideradas com problemas elevados ou muito elevados pela escala de conduta, 23% pela escala emocional, 15% pela escala de problemas com colegas, 13% pela escala de hiperatividade e 13% (baixo ou muito baixo) pela escala de comportamento pró-social. A escala total de dificuldades considerou 18% das crianças com problemas elevados ou muito elevados. No entanto, os números diferiram consideravelmente dos percentuais quando os pontos de corte baseados nos percentis amostrais do estudo foram usados. Além disso, nossos resultados mostraram que as escalas do SDQ apresentaram consistência interna modesta ou substancial em pré-escolares amazônicos.

Como esperado, nossos resultados diferiram de estudos realizados com crianças em outros países utilizando os mesmos pontos de corte. Um estudo chinês com 950 pré-escolares com idades entre três e cinco anos observou que 39% dos participantes tinham problemas emocionais; 27%, problemas de conduta; e 23%, problemas de hiperatividade^20^, todos superiores aos nossos resultados. No entanto, um estudo com 800 crianças paquistanesas com idades entre seis e 16 anos mostrou que 23% de sua amostra apresentou problemas emocionais; 27%, problemas de conduta; 11%, hiperatividade; 13%, problemas com colegas; 3% baixo ou muito baixo comportamento pró-social; e 16%, total de dificuldades comportamentais^21^.

No geral, comparando nossos resultados com os de outros estudos conduzidos no Brasil antes da pandemia de covid-19, os participantes da coorte MINA-Brasil mostraram maiores distúrbios comportamentais. Dados de uma coorte do sul do Brasil com 3.718 crianças mostraram que, aos seis anos de idade, 14,7% (16,1% meninos; 13,1% meninas) e 13,5% (13,0% meninos; 14,0% meninas) apresentavam níveis elevados ou muito elevados de conduta e problemas emocionais, respectivamente^22^. Outro estudo realizado no sul do Brasil com 152 escolares de seis a 13 anos observou maiores problemas de comportamento em crianças que viviam próximas a postos de reciclagem expostos ao lixo urbano do que naquelas que viviam a mais de 150 metros de distância^23^. Nesse estudo, os autores definiram os casos como crianças cujos problemas de comportamento foram limítrofes e anormais (equivalente à soma das classificações "ligeiramente elevada", "elevada" e "muito elevada" neste estudo). A frequência de casos diferiu entre as crianças para totais de dificuldades (24% no grupo exposto vs. 10% no grupo controle) e foi inferior aos dados do MINA-Brasil, que foi de 33,1% com base nos mesmos pontos de corte. No entanto, para algumas das outras subescalas do SDQ, nossos resultados foram inferiores aos do grupo controle (ex, subescalas hiperatividade e problemas com colegas).

Ao usar o modelo mais amplo de problemas internalizantes e externalizantes de comportamento, também observamos diferenças entre nossos resultados e os de outros estudos brasileiros antes da pandemia de covid-19. Dados de um estudo transversal na região sudeste do Brasil com 825 crianças de seis a 11 anos mostraram maior frequência de problemas internalizantes (30,7%) do que de externalizantes (18,3%)^24^, em contraste aos nossos resultados (16 e 17% para problemas internalizantes e externalizantes, respectivamente, na amostra MINA-Brasil).

Neste estudo, o SDQ foi aplicado durante a pandemia de covid-19, que causou direta ou indiretamente danos sem precedentes a toda a população, mas especialmente à primeira infância, uma vez que esta representa um período vulnerável na vida dos indivíduos. Embora as crianças não sejam consideradas um grupo de risco para a infecção pelo SARS-CoV-2, a pandemia agravou fatores comprometedores do desenvolvimento infantil, como estresse dos pais, suspensão das atividades escolares, isolamento social, falta de atividade física e insegurança alimentar, particularmente em contextos desestruturados^25^. A crise da covid-19 no Brasil foi ainda mais dramática na Amazônia, onde as unidades de saúde pública já estavam operando perto da capacidade total antes da pandemia^11^. Um estudo italiano com 463 crianças de cinco a 17 anos mostrou que as medidas de confinamento e as mudanças na rotina diária durante a pandemia de covid-19 afetaram negativamente as dimensões psicológicas dos pais, expondo as crianças a um risco significativo para seu bem-estar^26^. Isso pode explicar a maior frequência de problemas de comportamento em nosso estudo do que o esperado por outros estudos nacionais e internacionais.

Observamos diferenças de frequência ao comparar a proporção de crianças com problemas de comportamento de acordo com os pontos de corte originais do SDQ e aqueles elaborados neste estudo com base em nossa distribuição populacional. Em concordância, estudos recentes têm sugerido que o uso de normas europeias em diferentes contextos pode não refletir as realidades locais. Dados de diferentes continentes sustentam que, para fins clínicos, a definição de casos deve ser feita a partir de pontos de corte internos ao país^4^.

Com base em oito estudos de base populacional de sete países (Bangladesh, Brasil, Grã-Bretanha, Índia, Noruega, Rússia e Iêmen) com dados de mais de 29.225 crianças de cinco a 16 anos, os autores concluíram que as diferenças entre países no SDQ não refletem necessariamente diferenças comparáveis nas taxas de transtorno^27^. Portanto, interpretar frequências e outros resultados de comparações transculturais de saúde mental requer cautela, especialmente quando as avaliações se baseiam em questionários breves como o SDQ^27^.

Para investigar a medida de múltiplos itens que compõem um construto, muitos estudos sobre problemas sociais e comportamentais adotaram o alfa de Cronbach, inclusive utilizando o SDQ-P. No entanto, o alfa de Cronbach pode subestimar a verdadeira confiabilidade de um construto. Por isso, o ômega de McDonald tem sido cada vez mais adotado e relatado^28^. Embora muitos estudos do SDQ não tenham encontrado diferenças significativas entre os dois coeficientes, alguns autores recomendam o uso do ômega de McDonald^19^. Nossos resultados estão de acordo com estudos anteriores. Observamos coeficientes ômega de McDonald mais elevados do que os coeficientes alfa de Cronbach para a maioria das escalas do SDQ, inclusive quando estratificadas por sexo.

Nossa análise de consistência interna apoiou a utilidade potencial do SDQ no cenário deste estudo. Os resultados mostraram confiabilidade interna moderada para todas as escalas (0,60 ≤ α ≤ 0,40), exceto para a escala total de dificuldades, cuja confiabilidade foi substancial (α > 0,61). Um estudo com crianças brasileiras de seis e 11 anos encontrou resultados semelhantes: Os alfas de Cronbach variaram entre 0,59–0,65 e 0,52–0,59 para as subescalas de problemas de conduta e emocionais, respectivamente^22^.

Em concordância com outros estudos, nossa análise mostrou que a escala de problemas com colegas apresentou a pior consistência interna. Uma revisão de 26 estudos mostrou que os itens da escala de problemas com os colegas podem não refletir o mesmo construto, já que seus alfas foram os mais baixos para o SDQ^29^.

### Pontos fortes e limitações

Este estudo contribui significativamente para o progresso da ciência acerca da saúde mental na primeira infância na Amazônia durante as restrições covid-19. Isso ajuda a compreender as limitações e o potencial do SDQ, um instrumento de triagem de baixo custo e fácil aplicação. Vale ressaltar que, como dados sobre problemas de comportamento na infância no Norte do Brasil são raros, comparações requerem interpretações cautelosas. Analisamos dados de uma grande coorte de nascimentos de base populacional utilizando instrumentos validados que atribuíram credibilidade aos nossos achados. Entretanto, algumas limitações importantes deste estudo devem ser consideradas. Estimamos os pontos de corte do SDQ-P com base na distribuição de nossa própria população de estudo, que inclui uma faixa etária estreita (58,5 a 71,9 meses) e tem características socioeconômicas e culturais muito singulares. Assim, os resultados não devem ser replicados em outras populações. Além disso, embora nossas análises de consistência interna tenham apoiado a utilidade do SDQ em nossa população, reconhecemos a existência de diferentes possibilidades de análises a fim de explorar outras características psicométricas importantes de um instrumento^30^, que não foram o foco deste estudo. Dos participantes originais do MINA-Brasil recrutados ao nascimento (n = 1.240), a avaliação de cinco anos teve uma taxa de retenção de 56% (n = 695). As famílias das crianças que foram perdidas durante o seguimento da coorte tinham menor nível socioeconômico e escolaridade materna do que aquelas que participaram do acompanhamento de cinco anos. Assim, como a literatura aponta que um contexto de vulnerabilidade social se correlaciona com o mau desenvolvimento infantil, as frequências de distúrbios de comportamento relatadas neste estudo poderiam ser ainda maiores.

## CONCLUSÃO

Nossos resultados sobre problemas de comportamento infantil reforçam a necessidade de cuidados com a saúde mental no início da vida na região da Amazônia. Além disso, nossos resultados de consistência interna estão de acordo com os de outros estudos nacionais, apoiando a utilidade do SDQ. No entanto, interpretar nossos resultados e compará-los com os de outros estudos requer cautela devido à diversidade cultural e contextual em diferentes cenários.
